# The formation of hybrid complexes between isoenzymes of glyceraldehyde‐3‐phosphate dehydrogenase regulates its aggregation state, the glycolytic activity and sphingolipid status in *Saccharomyces cerevisiae*

**DOI:** 10.1111/1751-7915.13513

**Published:** 2019-11-19

**Authors:** Francisca Randez‐Gil, Isabel E. Sánchez‐Adriá, Francisco Estruch, Jose A. Prieto

**Affiliations:** ^1^ Department of Biotechnology Instituto de Agroquímica y Tecnología de los Alimentos Consejo Superior de Investigaciones Científicas Avda. Agustín Escardino, 7. Paterna 46980 Valencia Spain; ^2^ Departament of Biochemistry and Molecular Biology Universitat de València Dr. Moliner 50 Burjassot 46100 Spain

## Abstract

The glycolytic enzyme glyceraldehyde‐3‐phosphate dehydrogenase (GAPDH) has been traditionally considered a housekeeping protein involved in energy generation. However, evidence indicates that GAPDHs from different origins are tightly regulated and that this regulation may be on the basis of glycolysis‐related and glycolysis‐unrelated functions. In *Saccharomyces cerevisiae*, Tdh3 is the main GAPDH, although two other isoenzymes encoded by *TDH1* and *TDH2* have been identified. Like other GAPDHs, Tdh3 exists predominantly as a tetramer, although dimeric and monomeric forms have also been isolated. Mechanisms of Tdh3 regulation may thus imply changes in its oligomeric state or be based in its ability to interact with Tdh1 and/or Tdh2 to form hybrid complexes. However, no direct evidence of the existence of these interactions has been provided and the exact function of Tdh1,2 is unknown. Here, we show that Tdh1,2 immunopurified with a GFP‐tagged version of Tdh3 and that lack of this interaction stimulates the Tdh3’s aggregation. Furthermore, we found that the combined knockout of *TDH1* and *TDH2* promotes the loss of cell’s viability and increases the growing rate, glucose consumption and CO_2_ production, suggesting a higher glycolytic flux in the mutant cells. Consistent with this, the *tdh3* strain, which displays impaired *in vitro* GAPDH activity, exhibited the opposite phenotypes. Quite remarkably, *tdh1 tdh2* mutant cells show increased sensitivity to aureobasidin A, an inhibitor of the inositolphosphoryl ceramide synthase, while cells lacking Tdh3 showed improved tolerance. The results are in agreement with a link between glycolysis and sphingolipid (SLs) metabolism. Engineering Tdh activity could be thus exploited to alter the SLs status with consequences in different aspects of yeast biotechnology.

## Graphical abstract

The yeast isoenzymes Tdh1,2 physically interact with Tdh3, and regulate the Tdh3‐mediated GAPDH activity. A link between glycolysis and sphingolipid metabolism exists in *Saccharomyces cerevisiae*. Engineering Tdh activity could be exploited to alter the sphingolipid status with consequences in different aspects of yeast biotechnology.

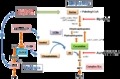

## Introduction

Energy‐generating metabolic pathways have a great influence on cell physiology, and consequently understanding the mechanisms of their regulation is of major interest. Metabolic activities of yeast cells rely primarily on the glycolytic pathway, which ensures ATP for energy and a high yield of product formation in biotechnological applications. Increased glucose metabolism through aerobic glycolysis is also one of the distinguishing features between normal cells and highly proliferating cells like cancer, stem and immune mammalian cells (Lunt and Vander Heiden, 2011). Targeting glycolysis regulation offers thus new opportunities to improve industrial and therapeutic outcomes.

The enzymes in glycolysis have a highly heterogeneous nature. Glycolytic isoenzymes are transcribed from different loci or are the result of different splice‐forms. In addition, they display a wide variety of expression rates and post‐translational modifications according to proliferation phase and environment (Warmoes and Locasale, 2014). These characteristics contribute to endow the pathway of the required flexibility to adjust the energy generation to the nutrient availability and the bioenergetics and biosynthetic demands. In *Saccharomyces cerevisiae*, Tdh3 is the main glyceraldehyde‐3‐phosphate dehydrogenase (GAPDH), although two other polypeptides encoded by *TDH1* and *TDH2* have been reported to exhibit GAPDH activity (McAlister and Holland, 1985). Like their mammalian counterparts, Tdh3 has been traditionally considered a housekeeping protein involved in energy generation. However, evidence indicates that GAPDH from different origins performs glycolysis‐unrelated functions (Zhang *et al.*, 2015; Muronetz *et al.*, 2017). Yeast Tdh3 has been described as a moonlighting protein (Gancedo *et al.*, 2016), as it facilitates the transcriptional silencing at the telomeres independently of its metabolic activity (Ringel *et al.*, 2013). This suggests that Tdh3 must be tightly regulated and that this regulation may be on the basis of its role in unrelated biological processes. For example, downregulation of Tdh3 activity has proven important to counteracting oxidative stress by rerouting the carbohydrate flux from glycolysis to the pentose phosphate pathway (Ralser *et al.*, 2007) and several post‐translational modifications have been mapped in the yeast enzyme (Halim *et al.*, 2015). Nevertheless, the impact of these modifications, the existence of additional Tdh3’s regulatory mechanisms and the exact function of Tdh1 and Tdh2 remain unclear.

Mechanisms of Tdh3 regulation may be based in its ability to interact with other proteins or imply changes in its oligomeric state. It is well known the presence of mammalian GAPDH in complexes and aggregates of different proteins (Sirover, 2011); including pathogenic proteins involved in various neurodegenerative disorders (Muronetz *et al.*, 2017). GAPDHs exist predominantly as a tetramer, although dimeric and monomeric forms have also been isolated (Sirover, 1999). GAPDH self‐aggregation induced by different conditions, i.e. oxidative stress, and regulated by post‐translational modifications, has been extensively reported (Nakajima *et al.*, 2009). In addition, Tdh3 could also interact with Tdh1 and/or Tdh2 to form hybrid tetramers, as it has been previously suggested (McAlister and Holland, 1985). This regulation may contribute to adjust the GAPDH activity and modulate the energy generation and the production of protective molecules according to the growth phase or under stress conditions. However, no direct evidence of the existence of these interactions has been provided and their significance has been not clarified.

Here, we show for the first time evidence of the physical interaction between Tdh isoenzymes, its importance in the stability of Tdh3 and the influence of Tdh1,2 on the glycolytic activity and the yeast lipid metabolism.

## Results and discussion

### Tdh3 forms heteromeric complexes with Tdh1,2 that localize preferentially in the soluble cytosolic fraction

**Figure 1:**
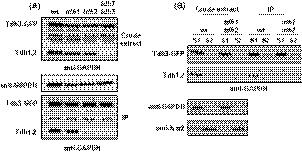
Tdh1,2 form mixed complexes with Tdh3. A. Protein crude extracts and anti‐GFP‐immunopurified (IP) samples from TDH3‐GFP transformants of the BY4741 wild‐type strain (wt) and its corresponding *tdh1*, *tdh2* and *tdh1 tdh2* mutants were analysed by Western blot. Tdh3‐GFP and Tdh1,2 were visualized with anti‐GAPDH. Glucose 6‐phosphate dehydrogenase (G6PDH) was used as a loading control. B. Protein fractions, S1 (soluble protein fraction) and S2 (membrane protein‐enriched fraction) from crude extracts and anti‐GFP‐immunopurifed (IP) samples of YPD‐grown cultures (OD_600_ ~ 0.5) of TDH3‐GFP transformants of wild‐type (wt) and *tdh1 tdh2* cells were analysed as in panel (A). Glucose 6‐phosphate dehydrogenase (G6PDH) and Kar2 were used as a loading control.

Co‐immunopurification experiments were conducted to analyse the presence of mixed complexes between Tdh1‐3 isoenzymes. The extensive sequence homology showed by the three forms of GAPDH activity in yeast does not allow the specific detection of any of them with polyclonal anti‐GAPDH antibodies. Therefore, we carry out a chromosomal tagging of Tdh3 at its C‐terminus with the GFP label. The addition of this tag increases the molecular weight of Tdh3 by 28.7 kDa, which allows its electrophoretic separation from Tdh1 and Tdh2, making possible its specific detection (simultaneously with Tdh1,2) using anti‐GAPH antibodies. In addition, the presence of the GFP tag allows the *in vivo* cellular localization of Tdh3‐GFP, as well as its efficient immunoprecipitation using anti‐GFP antibodies. Protein extracts from wild‐type, *tdh1*, *tdh2* and *tdh1 tdh2* strains containing a chromosomal copy of GFP‐tagged *TDH3* were resolved by SDS–PAGE and visualized by Western blot using an anti‐GAPDH antibody. As shown in Fig. 1A (upper panel), two major bands corresponding with Tdh3‐GFP (apparent Mw ~ 65 kDa) and Tdh1,2 (apparent Mw ~ 36 kDa) were observed in all the strains analysed, except for the *tdh1 tdh2* double mutant, where the higher‐mobility band was absent. Accordingly, the Tdh3‐GFP protein in the lysates was captured with anti‐GFP antibody and the resultant immune complexes analysed again by Western blot. As shown in Fig. 1A (lower panel; IP), the presence of a Tdh1,2‐band was again evident in protein samples from wild‐type, *tdh1* and *tdh2* cells. Moreover, we observed a weaker signal in the *tdh2* mutant samples, a result that is consistent with the low expression of *TDH1* in cells growing at the exponential phase, as previously reported (McAlister and Holland, 1985). Hence, we concluded that Tdh1,2 physically interacts with Tdh3.

Next, we analysed whether the interaction between Tdh isoenzymes could influence their subcellular localization. Protein extracts were fractionated by centrifugation, and cytosolic (S1) and membrane‐enriched (S2) fractions were analysed by SDS–PAGE before and after immunopurification with anti‐GFP antibody (Fig. 1B). As expected from a glycolytic enzyme, both Tdh3‐GFP and Tdh1,2 were found to be abundant in the soluble S1 fraction of wild‐type cells, although a significant portion of Tdh3‐GFP was also recovered in the particulate S2 sediment (Fig. 1B). On the contrary, Tdh1,2 was hardy visible in the S2 fraction. Consistent with this, hybrid complexes of Tdh3 and Tdh1,2 were only recovered from the S1‐immunoprecipitates (Fig. 1B; wt, IP panel). To check whether the distribution of Tdh3 between the S1 and S2 fraction was dependent on the presence of Tdh1,2, we performed the same experiment in the *tdh1 tdh2* double mutant strain. As shown in Fig. 1B, absence of Tdh1,2 did not modify the distribution of Tdh3‐GFP. Altogether, these data suggest that Tdh3, regardless of the presence of Tdh1,2, may form homooligomers that interact with the cellular membranes.

### Absence of Tdh1,2 stimulates Tdh3‐GFP aggregation in a growth‐phase specific manner

**Figure 2:**
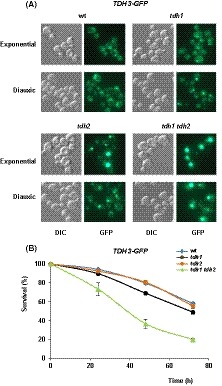
Lack of Tdh1 and Tdh2 influence cell viability and Tdh3‐aggregate formation. A. Wild‐type (wt), *tdh1*, *tdh2* and *tdh1 tdh2* mutant strains expressing Tdh3 fused to GFP were harvested in the early exponential phase or at the diauxic shift. Living cells were observed with a differential interference contrast (DIC) and by fluorescence microscopy (GFP). A representative experiment is shown. B. Strains shown in (A) were analysed for cell viability. The graph shows the percentage of surviving cells for 72 h of culturing in YPD medium. Details are given in the Experimental procedures section. Data represent the mean value (± SD) of three independent experiments.

We studied the cellular location of GFP‐tagged Tdh3 in wild‐type, *tdh1*, *tdh2* and *tdh1 tdh2* cells grown in the exponential phase (OD_600_ ~ 0.5) or at the diauxic shift (OD_600_ ~ 15.0). The fluorescence signal of Tdh3‐GFP was localized both in the cytoplasm and nucleus of the wild type (Fig. 2A). The simultaneous location of nuclei with DAPI or another dye was not possible due to the strong emission by Tdh3‐GFP and its interference in other microscope channels. However, in those cases where the formation of aggregates is low (Fig. 2A, in the wild‐type and *tdh1* mutant) it can be observed that the location of such aggregates does not coincide with the GFP signal that by position, morphology and size correspond to the fraction of Tdh3‐GFP located in the yeast nucleus. No apparent differences were observed in the Tdh3 distribution in relation to the growth phase (Fig. 2A). However, when Tdh1 was absent, the signal of Tdh3‐GFP in the diauxic‐shift cultures accumulated in fluorescence foci (Fig. 2A). In contrast, single *tdh2* mutant cells displayed focal fluorescence accumulation during their exponential growth, a pattern that gradually dissipated as cells began to enter the diauxic shift (Fig. 2A). These observations suggest that Tdh1 and Tdh2 play a role as molecular partners to stabilize Tdh3 and to avoid Tdh3‐aggregate formation along the growth curve. As earlier mentioned, *TDH2* and *TDH1* are expressed mainly in the exponential phase and when glucose becomes limiting respectively (McAlister and Holland, 1985). Consistent with this, the cells of the double *tdh1 tdh2* mutant strain exhibited Tdh3‐GFP fluorescence accumulation independently of the growth phase (Fig. 2A). The results support the idea that Tdh1,2 could play a role in preventing the aggregation of Tdh3 and/or its interaction with other proteins to form large complexes that would appear as fluorescent foci. Taking into account the results of the Tdh3 fractionation between the soluble and particulate fractions shown in Fig. 1B, we can conclude that the presence of a portion of Tdh3 in the particulate fraction is independent of the formation of these aggregates, since a part of Tdh3 was recovered into the particulate fraction in a wild‐type strain, where no aggregates are detected by fluorescence microscopy and, in addition, the amount of Tdh3 in the particulate fraction did not increase in the *tdh1 tdh2* double mutant strain (Fig. 1B) where large aggregates are detected (Fig. 2A).

### Effects on yeast viability

We wonder whether the interaction between Tdh1,2 and Tdh3 may represent a mechanism to regulate the Tdh3 function and as a result the yeast glycolysis. There is evidence that glycolytic isoenzymes provide complexity and heterogeneity to glycolysis in eukaryotic cells living either as interacting cell populations or as multicellular organisms (Warmoes and Locasale, 2014). To examine this possibility we first check whether the knockout of *TDH1*,*2* has any effect on yeast viability. Previous studies link the GAPDH activity with that of the Sir2 protein (Matecic *et al.*, 2002; Ralser *et al.*, 2009), a member of the sirtuins family (Schwer and Verdin, 2008), implied in replicative ageing and senescence in *S. cerevisiae* (Ha and Huh, 2011). It has been hypothesized that under high glycolytic rates, the action of GAPDH would reduce the cellular content of NAD^+^, depressing the Sir2‐mediated catalysis and decreasing lifespan (Ralser *et al.*, 2009). On the contrary, under low glycolytic activity the opposite would be expected to occur. As shown in Fig. 2B, deletion of *TDH1* or *TDH2* from wild‐type cells containing Tdh3‐GFP had no major effect on the survival of *S. cerevisiae*. However, the combined knockout of *TDH1* and *TDH2* caused a statistically significant (*P* < 0.01) reduction in viability (Fig. 2B).

### *tdh1* *tdh2* mutant cells show features of increased glycolysis

The above results suggested increased glycolysis in *tdh1 tdh2* mutant cells. To further examine this idea, we first measured the *in vitro* GAPDH activity in protein extracts from wild‐type, *tdh1*, *tdh2* and *tdh3* single mutant strains, and *tdh1 tdh2* and *tdh1 tdh3* double mutants. Cells lacking both Tdh2 and Tdh3 were not analysed because their poor growth. As expected, the loss of Tdh3 in the single *tdh3* or double *tdh1 tdh3* mutants caused a strong reduction of GAPDH activity (Table 1). The *TDH3* gene has been previously characterized as the major contributor to the yeast GAPDH activity (McAlister and Holland, 1985). However, neither the single disruption of *TDH1* nor that of *TDH2* caused apparent changes in the *in vitro* activity. Interestingly, the simultaneous lack of Tdh1 and Tdh2 seemed to increase slightly the catalysis mediated by Tdh3, but the variation was not statistically significant (Table 1).

**Table 1:** GAPDH activity in the strains under study.^a^

Strain	Units GAPDH/unit OD_600_ of cells
BY4741	5.78 ± 0.90
*tdh1*	5.74 ± 0.95
*tdh2*	5.86 ± 0.89
*tdh3*	2.00 ± 0.62*
*tdh1 tdh2*	6.16 ± 0.68
*tdh1 tdh3*	1.84 ± 0.48*

a The *in vitro* GAPDH of yeast cells of the BY4741 wild type, and its corresponding *tdh* mutant strains, was assayed as described in the Experimental procedures section. Data represent the mean value (± SD) of three independent experiments. Mutant samples denoted with * were significantly different from those of the wild type (*P* < 0.01).

**Figure 3:**
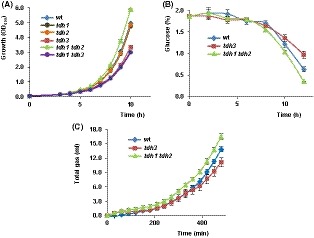
Initial logarithmic phase. A. Cells of the BY4741 wild type (wt) and its corresponding *tdh1*, *tdh2*, *tdh3*, *tdh1 tdh2* and *tdh1 tdh3* mutant strains were pre‐grown in liquid YPD medium, refreshed in the same medium (initial OD_600_ ~ 0.05), and their growth was followed for 10 h. Data represent the mean value (± SD) of two biological replicates. B. The mentioned strains, wild‐type (wt), *tdh3* and *tdh1 tdh2* were further analysed for glucose consumption. YPD culture medium was inoculated with yeast biomass and the residual glucose determined along the experiment as indicated in the Experimental procedures section. Values are the mean (± SD) of three independent experiments. C. YPD‐inoculated cultures of the indicated strains were assayed for gas production. The total gas accumulated (ml) during the time‐course of the experiment is shown. Data represent the mean value (± SD) of three independent experiments.

Then, we examined the growth of the mentioned strains in liquid YPD medium. Glycolysis generates biomass precursors and energy from sugars and is therefore a central element in the function of all anabolic pathways, and consequently, there is a tight coupling of cell growth to glycolysis (Lunt and Vander Heiden, 2011; Rehberg *et al.*, 2014). Cell pre‐cultures that have reached the stationary phase were diluted with fresh medium and the growth, as measured as OD_600_, was recorded during the initial logarithmic phase. As it is shown in Fig. 3A the onset of growth was similar for all the strains analysed. However, differences were soon evident. As expected, mutant cells lacking the GAPDH activity provided by Tdh3 displayed lower growth than the wild type (Fig. 3A). No effects on growth were observed by lacking Tdh1 or Tdh2, but the combined lack of both isoenzymes increased slightly cell proliferation (Fig. 3A). Consistent with this, glucose consumption (Fig. 3B) and total CO_2_ production (Fig. 3C) increased in the *tdh1 tdh2* double mutant as compared with the wild type, while the opposite behaviour was observed for the *tdh3* strain.

### Phenotypic characterization reveals an alteration of the sphingolipid metabolism induced by the Tdh‐isoenzymes profile

**Figure 4:**
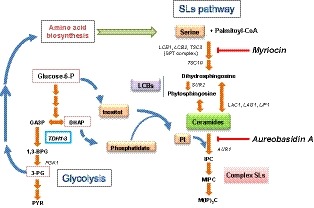
Schematic representation of the sphingolipid biosynthesis pathway in yeast and its main metabolic connections with glycolytic intermediates. Glucose carbon feeds the glycolytic pathway, which is mainly used to provide substrates of anabolic pathways. In this context, the sphingolipid synthesis is tightly linked to serine synthesis from 3‐phosphoglycerate (Vander Heiden *et al.*, 2009; Stathem *et al.*, 2015; Hwang *et al.*, 2017; Gao *et al.*, 2018). Only some glycolytic intermediates, genes involved and key metabolic steps for the discussion are represented. Additional information can be found in recent reviews (Henry *et al.*, 2012; Megyeri *et al.*, 2016; Olson *et al.*, 2016). Abbreviations: AUR1, aureobasidin A resistance, phosphatidylinositol:ceramide phosphoinositol transferase; 1,3‐BPG, 1,3‐bisphosphoglycerate; DHAP, dihydroxyacetone phosphate; Glucose 6‐P, glucose 6‐phosphate; GA3P, glyceraldehyde‐3‐phosphate; TDH1‐3, glyceraldehyde‐3‐phosphate dehydrogenase, isozyme 1‐3; IPC, inositolphosphoryl ceramide; LAC1, LAG1 cognate, ceramide synthase component; LAG1, longevity assurance gene, ceramide synthase component; LCBs, long‐chain bases; LCB1, long‐chain base 1, component of SPT complex; LCB2, long‐chain base 2, component of SPT complex; LIP1, Lag1/Lac1 interacting protein, ceramide synthase component; MIPC, mannosyl‐inositolphosphoryl ceramide; M(IP)2C, mannosyl‐di‐(inositolphosphoryl) ceramide; PI, phosphatidylinositol; 3‐PG, 3‐phosphoglycerate; PGK1, 3‐phosphoglycerate kinase; PYR, pyruvate; SPT complex, serine palmitoyltransferase complex; SUR2, sphinganine C4‐hydroxylase; TSC3, temperature‐sensitive suppressors of Csg2 mutants, component of SPT complex; TSC10, temperature‐sensitive suppressors of Csg2 mutants, 3‐ketosphinganine reductase.

Glycolysis is inefficient in terms of ATP production, as it compares with the oxidative phosphorylation (Berg *et al.*, 2007). Therefore, glycolytic metabolism in proliferating cells is mainly utilized to provide substrates of anabolic pathways, among them the sphingolipid (SLs) biosynthesis (Vander Heiden *et al.*, 2009), as it has been established in models of highly glycolytic cells (Stathem *et al.*, 2015). Indeed, different glycolytic intermediates, such as glucose 6‐phosphate, dihydroxyacetone phosphate and 3‐phosphoglycerate are precursors of inositol, phosphatidic acid and serine, which in turn, are substrates for the biosynthesis of long chain bases (LCBs), ceramides (Cer) and complex SLs classes in *S. cerevisiae* (Henry *et al.*, 2012; Megyeri *et al.*, 2016; Olson *et al.*, 2016; see Fig. 4). In this line, recent studies demonstrate that SLs and serine synthesis are tightly linked (Hwang *et al.*, 2017) and that serine availability influences cell proliferation (Gao *et al.*, 2018). Finally, several drugs that affect SLs metabolism can be easily used to estimate the impact of gene mutations in this anabolic branch. According to all of this, we investigated whether the loss of either Tdh1,2 or Tdh3 in wild‐type cells had an effect on SLs production.

**Figure 5:**
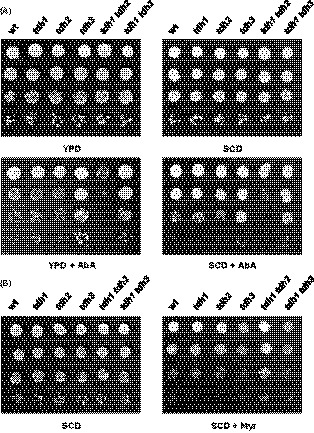
The profile of Tdh isoenzymes influences yeast sphingolipid status. A. Growth of the BY4741 strains, wild‐type (wt), *tdh1*, *tdh2*, *tdh3*, *tdh1 tdh2* and *tdh1 tdh3* was assayed in YPD or SCD medium in the absence or presence of 0.0675 μM aureobasidin A (AbA). B. The sensitivity to myriocin (Myr) of the same strains was checked in SCD medium containing 1.0 μM of the drug. Representative experiments are shown.

We first analysed the tolerance to aureobasidin A (AbA), a highly specific inhibitor of Aur1, the yeast inositolphosphoryl ceramide (IPC) synthase (Nagiec *et al.*, 1997; see Fig. 4). Exposure to AbA kills growing yeast cells (Endo *et al.*, 1997; Nagiec *et al.*, 1997), an effect that has been mainly related to the accumulation of toxic Cer. Increased Cer levels disturb membrane permeability, induce lethal mitophagy and trigger cell apoptosis (Sentelle *et al.*, 2012), and thus, cells deficient in SLs synthesis are resistant to AbA (Schorling *et al.*, 2001). On the contrary, increased Cer accumulation, i.e. by upregulation of LCBs biosynthesis, impaired ceramidase function and others, exacerbates the sensitivity of yeast cells to the drug (Vallée and Riezman, 2005). As shown in Fig. 5A, *tdh1* and *tdh2* single mutants grew on YPD or SCD plates containing AbA as did the wild‐type strain. Remarkably, cells of the *tdh3* and *tdh1 tdh3* mutants showed increased tolerance to the toxicity of AbA, while those of the *tdh1 tdh2* double mutant strain displayed a severe sensitivity in either YPD or SCD medium (Fig. 5A).

Then, we assessed the growth of the yeast strains under study in the presence of myriocin (Myr), an inhibitor of serine palmitoyltransferase, SPT (Wadsworth *et al.*, 2013), the enzyme complex that catalyses the first step in the SLs biosynthesis pathway (Megyeri *et al.*, 2016; Olson *et al.*, 2016). Unlike AbA, exposure to Myr reduces the levels of LCBs and Cer (see Fig. 4), and thus, cells sensitive to AbA are expected to be more tolerant to Myr and vice versa. As it is shown in Fig. 5B, cells harbouring a *TDH3* deletion were more sensitive to Myr than the wild‐type strain, although the phenotype was very subtle. We also observed than cells of the double *tdh1 tdh2* mutant grew better than those of the wild‐type or the single *tdh1* or *tdh2* mutants (Fig. 5B). Hence, the data suggest that the synthesis of SLs is stimulated in the *tdh1 tdh2* strain, while the loss of the main GAPDH isoenzyme Tdh3 depresses the SLs production.

## Concluding remarks

The combined evidences discussed herein indeed suggest that Tdh1 and Tdh2 participate in a concerted mechanism that functions to regulate the Tdh3‐mediated GAPDH activity. This mechanism could include changes in Tdh3’s cellular distribution according to carbon availability. Nevertheless, we were only able to show phenotypic changes when both isoenzymes Tdh1 and Tdh2 were absent, indicating some kind of functional overlapping. Interestingly, increasing evidence supports the notion that human GAPDH interacts with neurodegenerative disease‐related proteins, like α‐synuclein, by promoting the accumulation of oligomers and toxic aggregates (Ávila *et al.*, 2014; Mikhaylova *et al.*, 2016). The usefulness of *S. cerevisiae* as a model for the expression of human α‐synuclein showed by recent studies (Menezes *et al.*, 2015; Fruhmann *et al.*, 2017; Verbandt *et al.*, 2017) provide an interesting system to check whether Tdh1,2‐Tdh3 binding plays a role in the aggregation of this protein and the possibility of using yeast for the study of the mechanisms that underlie in the propagation of neurodegenerative diseases.

Our data indicate that Tdh1,2 contributes to maintain ATP and macromolecular biosynthesis as needed. Indeed, impaired growth on AbA and increased tolerance to Myr in *tdh1 tdh2* mutant cells are consistent with enhanced supply of serine and increased SLs metabolism. In addition, these features are exactly the opposites to those exhibited by cells deprived of Tdh3, which show a sharp reduction of *in vitro* GAPDH activity. Nevertheless, we cannot discard that some of these effects could be mediated, at least in part, by glycolysis‐unrelated functions. There is experimental evidence that Tdh3 is a moonlighting protein (Ringel *et al.*, 2013; Gancedo *et al.*, 2016), although more work is required to validate this in our experimental context. Meanwhile, our study establishes a link between glycolysis and SLs metabolism, and identifies GAPDH isoenzymes as putative targets for strain manipulation. Understanding how these and other components of the carbon central pathway are regulated in response to stress conditions or in industrial environments may facilitate the optimization of biotechnological processes by providing strategies for metabolic engineering.

## Experimental procedures

### Strains, plasmids, media and culture conditions

The *S. cerevisiae* strains, oligonucleotides and plasmids used in this study are listed in the Supporting information section (Tables S1–S3). Yeast cells were cultured at 30ºC in YPD (1% yeast extract, 2% peptone and 2% glucose). SCD contained 2% glucose as a carbon source and the appropriate amino acid drop‐out mixture (Formedium, England). Mutant strains lacking Tdh1 and/or Tdh2 were constructed by PCR‐based gene replacement using the kanMX4, natMX4 and hphMX4 disruption modules contained in plasmids pFA6a‐kanMX4 (Wach *et al*., 2004), pAG25 or pAG32 (Goldstein and McCusker, 1999), respectively (Table S3), and synthetic oligonucleotides (Table S2). Gene disruptions were confirmed by diagnostic PCR (Table S2). *S. cerevisiae* strains were transformed as previously described (Rodríguez‐Vargas *et al.*, 2007).

For plate phenotype experiments, cells were grown to the mid‐exponential phase at 30ºC (OD_600_ ~ 0.5). Then, 10‐fold serial dilutions were prepared and 3 μl aliquots of three dilutions (10–10^3^) were applied over the agar‐gelled plates. Colony growth was inspected after 2–4 days of incubation at 30ºC. Stock solutions of 1 mg ml^−1^ AbA (ethanol) and 2 mM Myr (ethanol:DMSO; 80:20, v:v) were prepared, sampled in small volumes and stored at −20ºC until use. For each experiment, a fresh sample was thawed and diluted at the indicated concentration.

### Preparation of protein extracts and Western blot analysis

Protein extracts, protein separation by SDS–PAGE and electroblotting were carried out as previously described (García‐Marqués *et al.*, 2016). A mouse‐raised antibody, which recognizes the GAPDH proteins (Delgado *et al.*, 2001) was kindly provided by D. Gozalbo. The antibody against Kar2 (1:10,000 dilution), a generous gift of J.L. Brodsky, and a commercial anti‐G6PDH antibody (1:3,000; cat#8866; Cell Signaling, Danvers, MA, USA) were used as loading control of membrane and cytosolic proteins respectively. Tdh3‐GFP was also detected with a monoclonal anti‐GFP antibody (1:3,000; cat#11814460001; Roche, Mannheim, Germany). As a secondary antibody, a horseradish peroxidase‐conjugated goat anti‐rabbit (1:2000, cat# 7074; Cell Signaling) or a rabbit anti‐mouse (1:5,000, cat# P0260; Dako, Carpinteria, CA, USA) was used. Blots were developed by using a Pierce® ECL kit (ThermoFisher Scientific, Rockford, IL, USA). Images were captured with the Las‐1000 Plus imaging system (Fuji, Kyoto, Japan) and processed identically by using Photoshop CS5.1 (Adobe System, San José, CA, USA).

### Protein fractionation and co‐immunoprecipitation experiments

Cells expressing a chromosomal *TDH3‐GFP* allele were lysed in ice‐cold buffer (50 mM HEPES, pH 7.4, 50 mM NaCl), and the corresponding protein extracts were centrifuged at 500 x *g* for 5 min to remove unbroken cells and glass beads. The clear supernatants were recentrifuged at 17 900 *g* for 10 min. The supernatant (S1, the soluble protein fraction) was collected, and the membrane‐associated proteins and protein aggregates were extracted by incubating the pellet on ice for 30 min with the same volume of lysis buffer that contained 1% Triton X‐100 and 0.001% Na^+^‐deoxycholate. The protein extract was recentrifuged and the new supernatant (S2, the membrane protein‐enriched fraction) was recovered. Tdh3‐GFP was immunoprecipitated as described previously (García‐Marqués *et al.*, 2016) by using Dynabeads® Protein A/G (ThermoFisher Scientific) conjugated with an anti‐GFP mouse monoclonal antibody (3 µl), separated by SDS–PAGE and immunoblotted with anti‐GAPDH, as described above.

### Enzyme activity determinations

Glyceraldehyde‐3‐phosphate dehydrogenase activity (GAPDH) was determined spectrophotometrically (McAlister and Holland, 1985). All the assays were performed at 25ºC. One unit is defined as the amount of enzyme that transforms 1 nmole of NAD^+^ per minute and unit of OD_600_ under assay conditions. Values represent the mean (± SD) of three independent experiments.

### Gas production and glucose consumption measurements

The volume of gas produced by yeast cells was monitored in a Fermograph II apparatus (ATTO Co., Ltd., Tokyo, Japan). Fifty millilitres of YPD was inoculated with yeast biomass (initial OD_600_ ~ 0.025) and incubated at 30ºC with low shaking (80 rpm). CO_2_ production was recorded at 30 min intervals for 480 min. Values are expressed as total CO_2_ (ml) accumulated during the indicated time and represent the mean (± SD) of three independent experiments.

Glucose was assayed by the 3,5‐dinitrosalicylic acid (DNS) colorimetric method (Miller, 1959). Aliquots (250 μl) of the yeast suspension were removed over the course of the experiment, centrifuged at 3,000 × g for 2 min (4ºC), and the supernatants collected for further analysis with the DNS reagent. Equal amounts of yeast biomass (initial OD_600_ ~ 0.025) were tested in YPD medium. Values are expressed as the percentage of glucose in the culture medium (g per 100 ml) and represent the mean (± SD) of three independent replicates.

### Fluorescence microscopy

The yeast cells grown in the early exponential phase (OD_600_ ~ 0.5) or diauxic phase (OD_600_ ~ 15) were used. Chromosomal GFP‐tagged Tdh3 was analysed in the living cells grown on YPD. The aliquots (1.2 μl) of the cultures were placed onto microscope slides and covered with 18 x 18 mm coverslips. Samples were analysed under an Axioskop 2 Fluorescence Microscope (Zeiss Inc., Jena, Germany), and pictures were taken with a SPOT digital camera (Diagnostic Instruments Inc., Sterling Heights, MI, USA) at identical exposures and processed identically by using Photoshop CS5.1 (Adobe System).

### Viability assay

Yeast cultures were incubated overnight in SCD at 30ºC with adequate shacking. Then, cells were refreshed at OD_600_ ~ 0.1. This time point was considered time 0. Then, cultures were maintained under the same conditions for 24, 48 and 72 h. At each time, aliquots from the culture were harvested, washed, resuspended in PBS buffer and analysed for cell viability by using a flow cytometer Muse® Cell Analyser (Millipore, Darmstadt, Germany) and the ‘Count and Viability’ kit reagent (Millipore). Data are expressed as the percentage of survival and represent the mean (± SD) of three independent experiments.

### Statistical analysis

Sample averages were compared by a Student’s *t*‐test with the Excel software (Microsoft). The samples denoted with * were significantly different (*P* < 0.05).

## Conflict of interest

None declared.

## Supporting information


**Table S1**. *Saccharomyces cerevisiae *strains used in this study.
**Table S2**. Oligonucleotides used in this study.
**Table S3**. Plasmids used in this study.
