# Supplementary material for: The formation of hybrid complexes between isoenzymes of glyceraldehyde‐3‐phosphate dehydrogenase regulates its aggregation state, the glycolytic activity and sphingolipid status in Saccharomyces cerevisiae
Source: Microb Biotechnol. 2019 Nov 19;13(2):562–71. doi: 10.1111/1751-7915.13513 (PMC7017825; doi:10.1111/1751-7915.13513)
Supplement: Supplementary file 1 — Table S1. Saccharomyces cerevisiae strains used in this study. Table S2. Oligonucleotides used in this study. Table S3. Plasmids used in this study. [file MBT2-13-562-s001.docx]

**Supplementary information**

**The formation of hybrid complexes between isoenzymes of glyceraldehyde-3-phosphate dehydrogenase regulates its aggregation state, the glycolytic activity and sphingolipid status in *Saccharomyces cerevisiae***

**Francisca Randez-Gil^1^, Isabel E. Sánchez-Adriá^1^, Francisco Estruch^2^ and Jose A. Prieto^1^**

**^1^**Department of Biotechnology, Instituto de Agroquímica y Tecnología de los Alimentos, Consejo Superior de Investigaciones Científicas, Avda. Agustín Escardino, 7. 46980-Paterna, Valencia, Spain.

**^2^**Departament of Biochemistry and Molecular Biology, Universitat de València, Dr. Moliner 50, Burjassot 46100, Spain.

**Table S1.** *Saccharomyces cerevisiae* strains used in this study

**Table S2.** Oligonucleotides used in this study

**Table S3.** Plasmids used in this study

**Table S1.** *Saccharomyces cerevisiae* strains used in this study

| Strain | Genotype | Reference or source |
| --- | --- | --- |
| BY4741 | *MATa his3*Δ* leu2*Δ* met15*Δ *ura3*Δ | Euroscarf |
| BY4741 *tdh1*Δ | BY4741 *tdh1*Δ*::kanMX4* | This study |
| BY4741 *tdh2*Δ | BY4741 *tdh2*Δ*::kanMX4* | Euroscarf |
| BY4741 *tdh3*Δ | BY4741 *tdh3*Δ*::kanMX4* | Euroscarf |
| BY4741 *tdh1*Δ *tdh2*Δ | BY4741 *tdh2*Δ*::kanMX4 tdh1*Δ*::natMX4* | This study |
| BY4741 *tdh1*Δ *tdh3*Δ | BY4741 *tdh3*Δ*::kanMX4 tdh1*Δ*::natMX4* | This study |
| BY4741 TDH3-GFP | BY4741 *TDH3-GFP::HIS3MX6* | ThermoFisher |
| BY4741 TDH3-GFP *tdh1*Δ | BY4741 *TDH3-GFP::HIS3MX6 tdh1*Δ*::hphMX4* | This study |
| BY4741 TDH3-GFP *tdh2*Δ | BY4741 *TDH3-GFP::HIS3MX6 tdh2*Δ*::hphMX4* | This study |
| BY4741 TDH3-GFP *tdh1*Δ *tdh2*Δ | BY4741 *TDH3-GFP::HIS3MX6 tdh1*Δ*::kanMX4 tdh2*Δ*::hphMX4* | This study |

**Table S2.** Oligonucleotides used in this study

| Name | Sequence | Used for |
| --- | --- | --- |
| TDH1-K1 | AGCAGTAAGAGCTTGGTGATAATGACCAAAACTGGAGTCTCGTACGCTGCAGGTCGAC | Deletion *TDH1* |
| TDH1-K2 | GTAATATGTACTTATTTACGTCTTCACAAAATCTAGCAGAATCGATGAATTCGAGCTCG | Deletion *TDH1* |
| TDH1-V1 | TCTTAGGTGCATGCGACGG | Verification deletion *TDH1* |
| TDH2-K1 | ACGTACGAACACATACGTATGCTAATATGTGTTTTGATAGTCGTACGCTGCAGGTCGAC | Deletion *TDH2* |
| TDH2-K2 | ATAAACGTGTATATGAGATGTCATGAGCATGAATTATTAAATCGATGAATTCGAGCTCG | Deletion *TDH2* |
| TDH2-V1 | GCAGTTGTTGCAACGCAGC | Verification deletion *TDH2* |

**Table S3.** Plasmids used in this study

| Plasmid | Description | Source or reference |
| --- | --- | --- |
|  |  |  |
| pFA6a-kanMX4 | pFA-yeast plasmid containing the kanr gene, which provide resistance to the drug geneticine. kanMX4 cassette template | Wach *et al*. (2004) |
| pAG25 | pFA-yeast plasmid containing the *nat^r^* gene, which provide resistance to the drug nourseothricin. natMX4 cassette template | Goldstein and McCusker (1999) |
| pAG32 | pFA-yeast plasmid containing the *hph^r^* gene, which provide resistance to the drug hygromycin B. hphMX4 cassette template | Goldstein and McCusker (1999) |
|  |  |  |

Wach, A., Brachat, A., Pöhlmann, R., and Philippsen, P. (2004) New heterologous modules for classical or PCR-based gene disruptions in *Saccharomyces cerevisiae*. *Yeast* **10:** 1793-1808.

Goldstein, A.L., and McCusker, J.H. (1999) Three new dominant drug resistance cassettes for gene disruption in *Saccharomyces cerevisiae*. *Yeast* **15:** 1541-1553.
